
JOURNAL INFORMATION
==============================
NLM Title Abbreviation: ScientificWorldJournal
Journal ID: ScientificWorldJournal
NLM Journal ID: 101131163
Journal ID: TSWJ

The Scientific World Journal

ISSN: 2356-6140
EISSN: 1537-744X
Publisher: Wiley

ARTICLE INFORMATION
==============================
PMCID: PMC6084249
PMID: 30147543
DOI: 10.1100/tsw.2001.118
Article ID: 798027
Article version: 1
Subjects: Short Report

Aging Blunts the Effect of Nitric Oxide on Myocardial O2 Consumption

Weiss Harvey R. *
Lazar Michael J.
Tse James
Scholz Peter M.
Heart and Brain Circulation Laboratory, UMDNJ-Robert Wood Johnson Medical School, 675 Hoes Lane, Piscataway, NJ 08854, USA
*Harvey R. Weiss: hweiss@umdnj.edu
Publication date: 2001
Electronic publication date: 2001 Nov 14
Volume: 1
First page: 70
Last page: 70
Copyright: Copyright © 2001 Harvey R. Weiss et al.
Copyright year: 2001
License: This is an open access article distributed under the Creative Commons Attribution License, which permits unrestricted use, distribution, and reproduction in any medium, provided the original work is properly cited.
License URL: https://creativecommons.org/licenses/by/3.0/

Funding: National Institutes of Health AG17648

==============================
Miami *Nature Biotechnology* Short Reports
*TheScientificWorld* (2001) 1(S3), 70SR
ISSN 1532-2246; DOI 10.1100/TSW.2001.118

### AGING BLUNTS THE EFFECT OF NITRIC OXIDE ON MYOCARDIAL $O_2$ CONSUMPTION

Harvey R. Weiss*, Michael J. Lazar, James Tse and Peter M. Scholz

Heart and Brain Circulation Laboratory, UMDNJ-Robert Wood Johnson Medical School,
675 Hoes Lane, Piscataway, NJ 08854, USA
* hweiss@umdnj.edu

**INTRODUCTION.** Aging reduces the ability of the heart to perform work and utilize energy (1). The nitric oxide-cyclic GMP signal transduction system reduces metabolism and function of the heart (2,3). Nitric oxide's effects are decreased in coronary endothelium with age (4), but there is an increase in guanylyl cyclase message and protein (5). We tested the hypothesis that the "braking" action of the nitric oxide-cyclic GMP system was reduced in old hearts.

**METHODS.** Studies were conducted in 13 young (~6 months) and 14 old (>3 years) New Zealand anesthetized open-chest rabbits. Either vehicle or S-nitroso-N-acetyl-D,L-penicillamine (SNAP, $10^{-4}$ M, a nitric oxide donor) was applied to the left ventricle. Coronary blood flow (microspheres) and $O_2$ extraction (microspectrophotometry) were used to determine subepicardial (EPI) and subendocardial (ENDO) $O_2$ consumption. Wall thickening was determined ultrasonically. Cyclic GMP and guanylyl cyclase activity were determined.

**RESULTS.** Myocardial $O_2$ extraction, flow, $O_2$ consumption and wall thickening were comparable in young and old hearts. The EPI and ENDO $O_2$ consumption of the SNAP treated young hearts decreased significantly (>25%) compared to vehicle, Fig. 1 (left). However, SNAP had no significant effects on the $O_2$ consumption of old hearts. SNAP also decreased the % wall thickening in the young (18.0±1.7 to 13.4±1.6%), but not old hearts (14.5±0.9 to 11.4%). The basal cyclic GMP levels in the old hearts were greater (~70%) than those in young hearts, Fig. 1 (right). SNAP increased cyclic GMP in EPI and ENDO of young, but not old, hearts. Guanylyl cyclase activity was assessed in both the young and old hearts at baseline and after increasing concentrations of sodium nitroprusside. Guanylyl cyclase activity was elevated in the old rabbit hearts with 0.5 mM nitroprusside (131±12 vs. 80±12 pmol/min/mg protein).

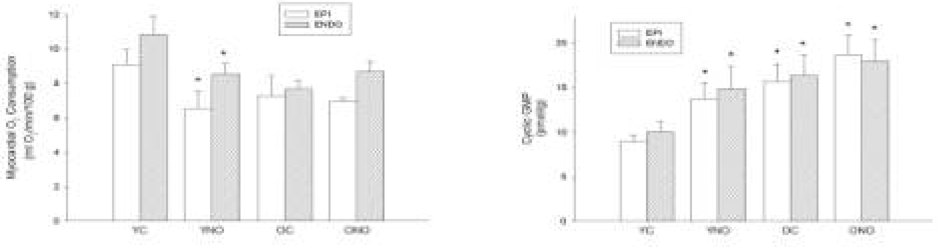

**Figure 1.** Myocardial $O_2$ consumption (left) and cyclic GMP (right) of young (YC) and old (OC) hearts treated with vehicle or young (YNO) and old (ONO) hearts treated with $10^{-4}$ M SNAP. * Significantly different from YC.

**DISCUSSION.** The major findings of this study were that while older hearts were metabolically and functionally similar to young hearts, they responded less well to nitric oxide and had significantly elevated basal levels of myocardial cyclic GMP. The effect of cyclic GMP is reduced in old endothelial cells (4). We found elevated levels of cyclic GMP and guanylyl cyclase activity. Guanylyl cyclase message and protein were increased with age (5). Thus, while cyclic GMP levels are elevated in older hearts, the response to the nitric oxide-cyclic GMP signal transduction system is depressed with age in heart.

**ACKNOWLEDGEMENT.** This work was supported by NIH Grant AG17648.

**REFERENCES.**

1. Kwong, L.K. and Sohal, R.S. (2000) Arch. Biochem. Biophys. 373, 16-22
2. Leone, R.J., Straznicka, M., Scholz, P.M., and Weiss, H.R. (2000) Basic Res. Cardiol. 95, 28-38
3. Straznicka, M., Gong, G., Yan, L., Scholz, P.M., and Weiss, H.R. (1999) J. Cardiovasc. Pharmacol. 34, 229-235
4. Chauhan, A., More, R.S., Mullins, P.A., Taylor, G., Petch, C., and Schofield, P.M. (1996) J. Am. Coll. Cardiol. 28, 1796-1804
5. Ruetten, H., Zabel, U., Linz, W., and Schmidt, H.H. (1999) Circ. Res. 85, 534-541
